# Complete mitogenomes of two major dengue vectors *Aedes aegypti* and *Aedes albopictus* from Bangladesh: Insights from comparative genomics with global mitogenome diversity and phylogenetics

**DOI:** 10.1371/journal.pone.0333693

**Published:** 2025-09-30

**Authors:** Md. Aminul Islam, Shefali Begum, Morjina Solaiman, Fatema-Tuz- Zohora, Sujan Kumar Datta, Khandaker Asif Ahmed, Md. Sagir Ahmed

**Affiliations:** 1 Entomology Laboratory, Department of Zoology, University of Dhaka, Dhaka, Bangladesh; 2 Advanced Fisheries and DNA Barcoding Laboratory, Department of Zoology, University of Dhaka, Dhaka, Bangladesh; 3 Department of Biological Sciences, University of Idaho, Moscow, Idaho, United States of America; 4 Department of Zoology, Jagannath University, Dhaka, Bangladesh; 5 CSIRO Australian Animal Health Laboratory (AAHL), East Geelong, Victoria, Australia; Saint Xavier's College, INDIA

## Abstract

*Aedes aegypti* and *Aedes albopictus* are the most competent vectors of multiple human arboviral diseases, posing a global public health concern, particularly in Bangladesh. While there are numerous genomic resources available worldwide, the genomic resources from Southeast Asian countries are scarce. Current study reported the first characterization of the complete mitochondrial (mt) genomes of *Ae. aegypti* and *Ae. albopictus* from Bangladesh. The circular mitogenomes are 16,662 bp and 16,585 bp in size, with AT contents of 79.02% and 78.81%, respectively. Both mitogenomes contain all 37 functional subunits, including 13 protein-coding genes (PCGs), two ribosomal RNA genes, 22 transfer RNA genes (tRNAs), and a control region. We also reported the unique codon usage and Relative synonymous codon usage patterns, which are congruent with other members of the Culicine family. Our intraspecific analysis of 15 mitochondrial genes revealed higher genetic distance, but lower SNP density in *Ae. aegypti*, compared to *Ae. albopictus*, indicating *Ae. aegypti* might have a longer evolutionary history, while *Ae. albopictus* might have experienced recent population expansion with steady divergence. Similarity metrics and phylogeny of 13 PCGs from 37 taxa indicated both species of *Aedes* formed specific clusters, where Bangladeshi *Aedes* spp*.* showed close relationships with North American, Oceania and European populations. Our comprehensive tree incorporating *Aedes, Ochleroratus and Culex* spp*.* revealed distinct clades, aligning with previous studies. Current study provides a foundation for future research on *Aedes* spp*.* of Bangladesh, leveraging it to other Southeast Asian countries, aiding vector identification, control, and disease mitigation.

## Introduction

The genus *Aedes* is medically significant, with species like *Aedes aegypti* (Linnaeus, 1762) and *Aedes albopictus* (Skuse, 1894) serving as vectors for several arboviruses that cause diseases like Dengue, Yellow Fever, Chikungunya, and Zika in humans. The native range of *Ae. aegypti* is West Africa, while *Ae. albopictus* originates from Southeast Asia, but both species are now found worldwide, including in the Indo-Pacific regions [1–3]. *Ae. ageypti* and *Ae. albopictus* are most prevalent in low-income countries, such as Bangladesh [4–6]. Geographically, Bangladesh is located at the center of South-East Asia, with a well-suited environment for mosquito breeding. Besides, wide distribution and strong adaptability, their diurnal biting habits and human blood preferences have contributed to the serial outbreaks of several mosquito-borne diseases in recent years [7–10]. Recently, Dengue fever has become a significant concern for public health, causing socioeconomic losses all over the country. Bangladesh has experienced sporadic outbreaks of Dengue since 1964, followed by the first large outbreak in 2000 [11]. The infection and deaths upsurged in outbreaks in the following 2019 and 2023 years, with >100,000 and >250,000 infected individuals and >160 and >1,700 deaths respectively [7,12].

To control the spread of Dengue and other mosquito-transmitting diseases, it is important to control mosquito populations, especially these two *Aedes* spp. [9,13]. Bangladesh has taken numerous unorganized strategies, e.g., insecticide spray, using mosquito nets in houses, campaigns for maintaining hygiene and destroying mosquito habitats, but as one of the most densely populated countries around the world, these measures are surely not enough [14]. Unorganized practices of insecticide usage often resulted in insecticide resistance. For example, one study reported widespread pyrethroid resistance in *Ae. aegypti* population from Bangladesh, with population-specific variabilities in deltamethrin and malathion susceptibilities [15]. A subsequent study on *Ae. albopictus* further demonstrated resistance to pyrethroid (permethrin), while showing susceptibility to pyrethroid (deltamethrin), organophosphate (malathion), and Carbamate (bendiocarb) insecticides [16]. For a comprehensive management program, it is important to understand the endemic populations, habitat characterization, and most importantly the unique genetic elements of local populations.

A review study reported 123 mosquito species from Bangladesh [17]. Although our literature search can find gene sequences (e.g., *cox1*, *16S* or microsatellites) of these species from various parts of the world [18–20], there is a scarcity of genomic resources of Dengue vectors from Southeast Asian countries, including Bangladesh. A morphological and partial *cox1*-based study by Siddiki *et al.,* (2019) [21] reported three mosquito species, namely *Ae. albopictus*, *Ae. aegypti* and *C. pipiens* from various locations of South-eastern part (Chattogram Metropolitan area) of Bangladesh. A recent study [22] has identified eleven species mosquitoes, including two species of *Aedes*, from six different locations from Bangladesh, using partial *cox1* gene sequences. Further, using two mitochondrial gene sequences [23] recorded few *C. pipiens* complexes from Gulshan, Dhaka, Bangladesh, reporting the first record of *C. pipiens pallens* in the Indian region, which is commonly found in East Asian countries and vectors of filariasis.

A partial mitochondrial sequence cannot confer the overall genetic markup of a species. To understand these two species comprehensively, it is important to look at the bigger picture, at the whole genome scale, or at least at the maternally inherited whole mitogenome level. Population level and high-resolution genetic studies are essential to control the spread of mosquito-borne diseases. Further, it can set up baseline resources to evaluate the efficacy of existing pesticide-based control strategies. It is intriguing that, even though there are several mosquito mitogenomes sequenced worldwide, none of them are from Southeast Asian countries, which are considered a hotspot of these mosquitoes. Also, existing mitogenomes show a high skew for the *Anopheles* genus; considerably less data is known for *Aedes, Ochlerotatus, Culex,* and other genera [24]. This gap in knowledge hinders our ability to apply genomic insights to local vector control efforts.

The present study aims to fill this gap by utilizing high thought put, Illumina short-read based sequencing technology and subsequently assembly and curation of the complete mitogenomes of these two *Aedes* spp. To our knowledge, this is the first record and detailed characterization of the complete mitochondrial genomes from Bangladesh, broadly Southeast Asia. We also conducted a comparative phylogenetic analysis with 37 other complete mitogenomes, reported in NCBI to date. Our detailed characterization and comparison with other *Aedes* spp. from different countries set up a baseline for future research of whole genome level comparison, and species uniqueness and will also play a vital role in the development of more effective strategies for dengue control in the region.

## Materials and methods

### Sample collection and identification

The specimen of adult *Ae. aegypti* individual was collected from Kamrangirchar, Dhaka, Bangladesh (geographic location: 23.71756194 N 90.37314583 E) on August 11, 2023, using a sweep net. On the other hand, *Ae. albopictus* was collected from Curzon Hall, Dhaka, Bangladesh (geographic location: 23.727000 N 90.401462 E) on August 14, 2023, using the same process. The collected specimens were identified using standard dichotomous taxonomic keys [25].

### Genomic DNA extraction and sequencing

Genomic DNA was extracted from both collected specimens using the DNeasy Blood & Tissue Kit (Qiagen, Germany) following the manufacturer’s protocol. The quality of DNA was assessed using a NanoDrop spectrophotometer (Thermo Fisher Scientific, USA). Only high-quality DNA samples were sent for sequencing. The library preparation and Illumina-based sequencing were commercially sourced at GENEWIZ Genomic Center, Suzhou, China. From the prepared library, 150 bp paired-end reads were generated and sequenced in a NovaSeq 6000 system (Illumina, San Diego, CA). A total of 10Gb of raw data per species was generated.

### Preprocessing and Bioinformatics Workflow

The sequences were processed as per previously described methods [26]. Briefly, the read quality was checked by FastQC (0.12.1) [27] and quality was trimmed using TrimGalore v6.6 [28]. Trimmed reads were used to assemble mitogenomes of *Ae. aegypti* and *Ae. albopictus*. Reference-based assemblies were conducted using CLC Genomics Workbench 23.0.4 (CLC Bio, Aarhus, Denmark), where respective complete mitogenomes (NC_035159.1 for *Ae. aegypti* and KR068634.1 for *Ae. albopictus*) were used as references. Assemblies were confirmed visually by mapping raw reads back to each assembly. For annotation, multiple sequence alignment was performed retrieving other mosquito mitogenomes from GenBank using MAFFT v7.2.1.5 [29]. The circular map of the mitogenomes of both species was drawn using the Geneious Programme. Base composition and skewness were calculated in Seqkit [30] and Microsoft Excel programs and plotted using ggplot2 R Package [31]. Relative Synonymous Codon Usage was calculated using the Codon Adaptation Index calculator (CAI) [32].

### Phylogenetic analysis

For phylogenetic tree construction, a total of 37 mitogenome sequences (two from this study, and 35 derived from GenBank) were considered. The species names and associated accession numbers are shown in the generated tree. For each species, all 13 protein-coding genes were concatenated, making a single sequence for each. The coding sequences were aligned using MAFFT v7.2.1.5 [29], followed by optimal model selection, a maximum likelihood (ML) based phylogeny, and bootstrap analysis using IQ-Tree [33]. The iTol web server was used to visualize the consensus cladogram [34]. Bootstrap values ≥70% were considered to represent moderate confidence [35]. Additionally, for Bayesian inferences, BEAST software suite (v.10.5.0) was utilized. Briefly, the aligned sequences were used to create an XML file with HKY substitution model, using BEAUTi module and phylogenetic analysis conducted using BEAST. The tree was constructed using TreeAnnotator module, with 1000000 Burnin and the target tree type as Maximum clade credibility tree. The resulting nexus file was visualized using iTol web server.

## Results

### Features of *Ae. aegypti* and *Ae. albopictus* mitogenomes from Bangladesh

**Fig 1:**
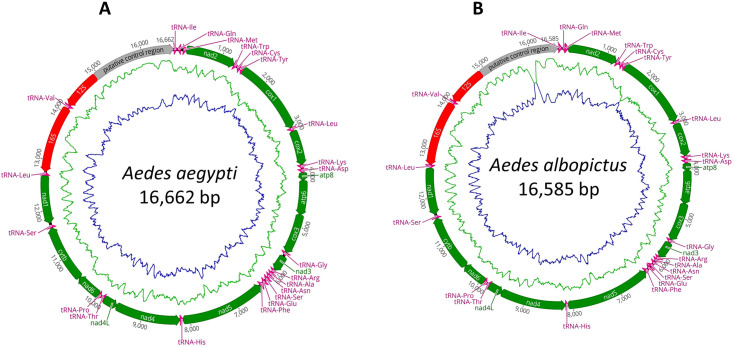
Circular mitochondrial genomes of (A) *Ae. aegypti* (B) *Ae. albopictus,* reported in current study.

The complete mitogenomes of *Ae. aegypti* and *Ae. albopictus* were determined to be 16,662 bp and 16,585 bp in length, respectively, with high sequence similarities (99.75% and 99.85%) to NCBI reference mitogenomes (**Fig 1**). The complete mitogenomes sequences were submitted to GenBank with accession number PQ197330, and PQ197331, respectively. Both mitogenomes were circular, containing all 37 expected genes including 13 protein-coding genes (PCGs; *cytb, atp6, atp8, nad1–6, cox1–3* and *nad4L*), 2 ribosomal RNAs (rRNAs) (*12S* and *16S*) and 22 tRNAs with a AT-rich control region (*CR*). Both species’ mitochondrial sequences exhibited AT-biasness. Overall, in both mitogenomes, GC content was 20.98 and 20.10% in *Ae. aegypti* and *Ae. albopictus*, respectively – but AT content was elevated to 78.02% and 79.02%, which are > 4 times higher than GC content. The AT skew values are 0.02 and 0.01, and the GC skew values are −0.22 and −0.19, respectively (S1 Fig).

Both mitogenomes contain 13 PCGs with variable gene lengths. The extent of the protein-coding gene (PCG) regions was 11,191 base pairs in *Ae. aegypti* and 11,223 base pairs in *Ae. Albopictus* (Table 1). The relative lengths of the PCGs (range:162–1731 bp) showed the subsequent organization*: nad5 > cox1 > nad4 > cytb> nad2 > nad1 > cox3 > cox2 > atp6 > nad6 > nad3 > nad4L > atp8*. In both sequences, nine PCGs, specifically *cox1, cox2, cox3, nad2, nad3, nad6 atp8, atp6,* and *cytb*, showed a sense of transcription on the forward strand (+), and the remaining PCGs, namely *nad5, nad4, nad4L,* and *nad1*, displayed the sense of transcription on the reverse strand (-). Most PCGs used ATN as the start codon, with *cox1* employing a noncanonical TCG start codon. Among the start codons, ATG was the most prevalent (in *cox2*, *atp6*, *cox3*, *nad4*, *nad4L*, and *cytb*), followed by ATT (in *nad2*, *atp8*, *nad3*, and *nad6*). On the other hand, the stop codons were predominantly TAA, although some incomplete stop codons (T or TA) were observed (**Table 1**).

**Table 1:** Nucleotide composition of *Ae. aegypti* and *Ae. albopictus* mitogenomes, reported in current study.

Gene	Strand	Position		Size		Intergenic nucleotides		Start/stop codon		Anticodon
	AA/AAlb	AA	AAlb	AA	AAlb		AA	AAlb	AA	AAlb
*trnI*	+/+	1-67	1-69	67	69	0	0			GAT
*trnQ*	-/-	70-138	79-138	69	60	−1	59			TTG
*trnM*	+/+	188-256	139-208	69	70	8	70			CAT
*nad2*	+/+	257-1282	208-1233	1026	1026	955	1017	ATT/TAA	ATT/TAA	
*trnW*	+/+	1283-1352	1234-1303	70	70	−957	−886			TCA
*trnC*	-/-	1352-1418	1303-1369	67	67	−4	1023			GCA
*trnY*	-/-	1419-1486	1369-1435	68	67	0	70			
*cox1*	+/+	1485-3021	1434-2970	1537	1537	1469	1536	TCG/T(AA)	TCG/T(AA)	
*trnL2*	+/+	3022-3088	2970-3038	67	69	−1471	−1401			TAA
*cox2*	+/+	3090-3774	3029-3723	685	685	615	2155	ATG/T(AA)	ATG/T(AA)	
*trnK*	+/+	3775-3845	3723-3795	71	73	−615	−543			CTT
*trnD*	+/+	3862-3930	3807-3875	69	69	−5	683			GTC
*atp8*	+/+	3931-4092	3876-4037	162	162	92	166	ATT/TAA	ATT/TAA	
*atp6*	+/+	4086-4766	4031-4711	681	681	518	588	ATG/TAA	ATG/TAA	
*cox3*	+/+	4770-5557	4715-5502	788	788	106	269	ATG/TA(A)	ATG/TA(A)	
*trnG*	+/+	5558-5624	5503-5569	67	67	−722	−40			TCC
*nad3*	+/+	5649-5977	5570-5923	329	354	261	1075	ATT/TA(A)	ATT/TAA	
*trnR*	+/+	5978-6037	5923-5985	60	63	−295	−199			
*trnA*	+/+	6042-6110	5986-6053	69	68	5	362			TGC
*trnN*	+/+	6112-6181	6055-6121	70	67	1	61			GTT
*trnS1*	+/-	6183-6249	6120-6186	67	67	−1	65			
*trnE*	+/+	6262-6327	6203-6270	66	68	−2	68			
*trnF*	-/-	6326-6392	6268-6334	67	67	−2	68			GAA
*nad5*	-/-	6400-8142	6342-8084	1743	1743	1675	1744	GTG/TAA	GTG/TAA	
*trnH*	-/-	8143-8208	8085-8150	66	66	−1678	−1610			
*nad4*	-/-	8214-9597	8153-9496	1344	1344	1277	3021	ATG/TAA	ATG/TAA	
*nad4L*	-/-	9551-9847	9490-9786	297	297	−1048	−981	ATG/TAA	ATG/TAA	
*trnT*	+/+	9850-9915	9789-9853	66	65	−232	1112			TGT
*trnP*	-/-	9916-9982	9854-9920	67	67	1	298			TGG
*nad6*	+/+	9985-10506	9920-10444	522	524	454	522	ATT/TAA	ATA/TAA	
*cytb*	+/+	10510-11644	10444-11580	1135	1137	610	682	ATG/T(AA)	ATG/TAA	
*trnS2*	+/+	11645-11710	11580-11645	66	66	−1072	−545			
*nad1*	-/-	11734-12675	11668-12612	942	945	875	2016	ATA/TAA	ATA/TAA	
*trnL1*	+/-	12685-12752	12619-12686	68	68	−878	−808			TAG
*rrnL*	-/-	12753-14087	12688-14021	1335	1334	1266	2211			
*trnV*	-/-	14089-14160	14023-14094	72	72	−1263	−1195			TAC
*rrnS*	-/-	14162-14941	14095-14891	780	797	707	2059			
*CR*	+/+	14942-16662	14892-16585	1721	1694	923	986			

AA=*Ae. aegypti*; AAlb *=Ae. albopictus*; CR = Control region.

**Fig 2:**
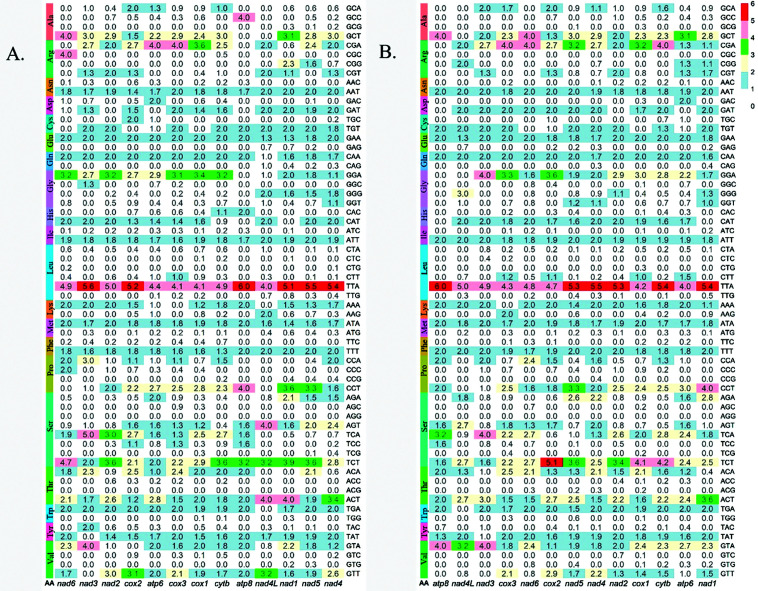
Relative synonymous codon usage (RSCU) of the 13 protein coding genes of (A) *Ae. aegypti* (B) *Ae. albopictus.*

Throughout the 13 protein-coding genes Serine (Ser) and Leucine were most recurrent exhibiting codon usages (11.67% each) and (10%) respectively. Serine with 7 distinct codons and Leucine with 6 codons. In both *Ae. aegypti* and *Ae. albopictus*, Proline (Pro), Alanine (Ala), Valine (Val), Glycine (Gly), and Arginine (Arg) utilized 4 different codons (6.67%) (**Fig 2**). Threonine (Thr) and Arginine (Arg) used 3 different codons (5%) in *Ae. aegypti* and *Ae. albopictus* (**Fig 2**), respectively. In both species, the remaining amino acids had codon usages of 3.34% utilizing only two codons each (**Fig 2**). Some codons were used preferentially across all PCGs, with *TTA (Leu)* showing the highest abundance (RSCU > 4). Codon usage bias was similar between the two species. Codon usage bias highlights evolutionary pressures shaping mitochondrial genomes. Among the 60 codons, 10 codons (GAA for glutamic acid, TTA for leucine, AAT for asparagine, ATA for methionine, CAA for glutamine, AGT and TCT for serine, ATT for isoleucine, TTT for phenylalanine, and ACT for threonine) in *Ae. aegypti*, while 12 codons (TTA for leucine, AAT for asparagine, ATA for methionine, AGT, TCA, and TCT for serine, ATT for isoleucine, TTT for phenylalanine, ACA and ACT for threonine, TAT for tyrosine, and GTA for valine) in *Ae. albopictus* emerged throughout the 13 PCGs (RSCU > 0). The TTA codon of leucine had an RSCU value greater than 4 across all 13 PCGs, indicating its highest abundance in both species. Additionally, summing up the RSCU values across the PCGs, the TTA codon of leucine had higher cumulative RSCU values of 64.20 in *Ae. aegypti* and 64.80 in *Ae. albopictus*. Conversely, low RSCU values, less than 2, were observed in six codons in *Ae. aegypti* and eight codons in *Ae. albopictus* across the 13 PCGs (**Fig 2**). The codon ACG for threonine was used in *Ae. albopictus* but absent in *Ae. aegypti* (**Fig 2B**), whereas CGC for arginine was used in *Ae. aegypti* but unused in *Ae. albopictus* (**Fig 2A**).

The placement of the *12S* and *16S* (ribosomal RNA genes) was consistent across both mt genomes, whereas *16S* (large subunit) was surrounded by tRNA-Leu and tRNA-Val, which were 1,333 bp (*Ae. aegypti*) and 1,332 bp (*Ae. albopictus*) in lengths and *12S* (small subunit) was flanked by tRNA-Val and the AT-rich region, which were 778 bp (*Ae. aegypti*) and 795 bp (*Ae. albopictus*) long. Individually, the length of the large ribosomal subunit *16S* had narrow variation whereas the small ribosomal subunit *12S* was a little bit spacious. The AT-rich CR region is one of the highlights of both assemblies. We found the CR of *Ae. aegypti* is larger (1,721 bp), compared to that of *Ae. albopictus* (1,694).

### Evolutionary relationship of *Aedes* species from different countries

**Fig 3:**
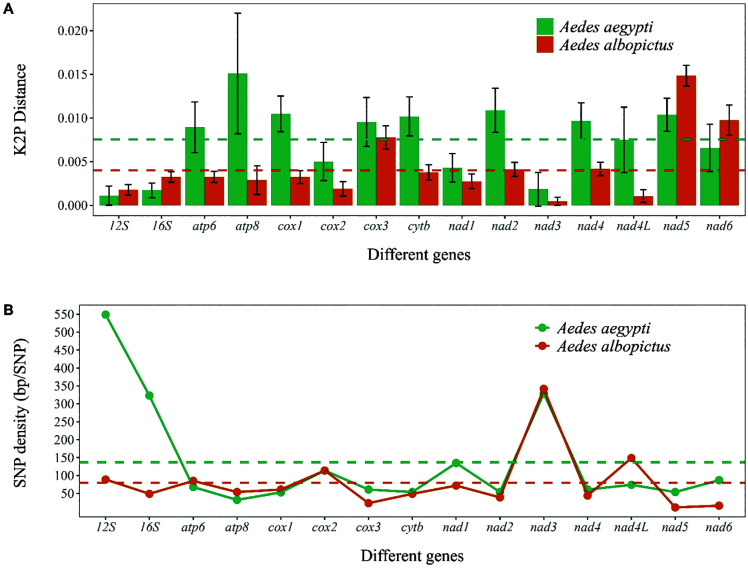
(A) K2P distance and (B) SNP density across 13 PCGs and two rRNAs of *Ae. aegypti* and *Ae. albopictus.*

We compared gene sequences from various countries, including six *Aedes aegypti* and thirteen *Aedes albopictus* specimens. We considered 13 PCGs and 2 rRNA sequences. As the CR region is not well annotated in several NCBI records, we did not consider it for this analysis. In terms of K2P distance, we found an average distance of 0.008 and 0.004 (dotted lines in **Fig 3A**) in *Ae. aegypti* and *Ae. albopictus* respectively, showing higher genetic diversity of mitochondrial genes of *Ae. aegypti* than that of *Ae. albopictus*. In *Ae. aegypti*, *atp8,* and *16S* genes showed the highest and lowest distances, while in *Ae. albopictus*, *nad5,* and *nad3* showed the highest and lowest values. Interestingly, K2P distances of two rRNAs (*12S* and *16S*) and two *nad*-genes (*nad5* and *nad6*) were less in *Ae. aegypti*, compared to other species. All other PCGs showed high distance across *Ae. aegypti*. The large error bars across *Ae. aegypti* genes indicate higher within-species genetic variations, including a possibility of greater evolutionary divergence within populations around the world.

The base pair per single nucleotide polymorphism (SNP) is shown in **Fig 3B**. A mean base pair per SNP of 137 and 80 bp is found in *Ae. aegypti* and *Ae. albopictus* genes, which means one SNP variation was detected in every 137 and 80 bp of these species respectively. Overall, *Ae. aegypti* showed high estimates, especially in *rRNA* genes, demonstrating high conservation of these gene sequences across all specimens considered. On the contrary, relatively stable estimates were found across all the PCGs and *rRNA* genes in *Ae. albopictus,* which conveys uniform mutational distribution. At the gene level, the highest and lowest estimates were observed in *12S* (549) *atp8* (32), and *nad3* (342) *and nad5* (11) genes, in *Ae. aegypti* and *Ae. albopictus*, respectively. Interestingly, both species showed the same values for *cox2*, *cytb*, *nad2,* and *nad3* genes. By combining insights from K2P and base pair per SNP estimates, our analysis showed some interesting insights. We have found higher K2P distance and base pair per SNP (fewer SNPs) in *Ae. aegypti*. It can be due to mutation clustering in specific genes, rather than evenly spread out across the genes. This may convey toward longer evolutionary history with isolated populations, where mutations could accumulate over time, increasing genetic distance, but conserving few SNPs per gene.

### Phylogeny across various mosquito mitogenomes

A similarity matrix and phylogenetic tree based on all 13 protein-coding regions, were generated to find the position of *Ae. aegypti* and *Ae. albopictus* among other mitogenomes available at NCBI (S1 Table). The identity matrix revealed that, *Ae. aegypti* from Bangladesh exhibited high similarities with 99.7% and 99.8% identities to populations from Australia and Trinidad-Tobago (S2 Fig). The similarity with the United Kingdom and Brazil was slightly lower at 98.6%. In contrast, *Ae. albopictus* showed remarkable similarity, with a 99.9% identity with several countries, including Thailand, the USA, Cameroon, Albania, Greece, Italy, Mexico, Japan, France, the Philippines, Brazil, and Taiwan.

**Fig 4:**
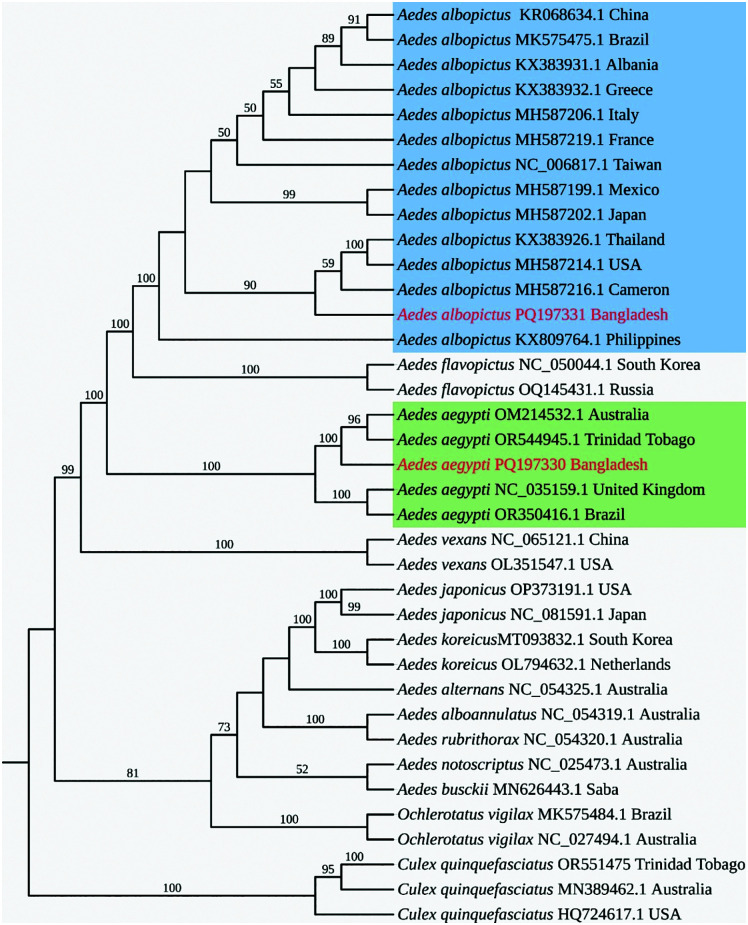
A Phylogenetic tree showing different mosquito mitogenomes. The maximum likelihood tree was constructed by concatenation of 13 PCGs listed in *Supplementary* S1 Table. 1000 bootstrap values were considered. The species sequenced in this study are shown in red.

Using the concatenated PCG, we have constructed a ML tree (Fig 4) and a Bayesian inference tree (S3 Fig), which showed mostly similar topology with higher Bootstrap (>75%) and Bayesian posterior probability ≥ 0.95. In the phylogenetic tree, *Ae. aegypti* and *Ae. albopictus* are positioned within distinct clades. *Ae. aegypti* from Bangladesh clustered with specimen from Australia and Trinidad-Tobago with a bootstrap value of 96–100%, while *Ae. albopictus* formed clades with Thailand, USA, and Cameroon populations (90–100%). The evolutionary divergence of *Ae. aegypti* and *Ae. albopictus* from their common ancestor was confirmed, with outgroup *C. quinquefasciatus* forming a distinct outgroup. Phylogenetic analysis showed that the *Ae. aegypti* mitogenome from Bangladesh clusters with its counterparts from Australia and Trinidad and Tobago, forming a clade. Meanwhile, *Ae. aegypti* from the United Kingdom and Brazil form a separate clade with 100% bootstrap support. Similarly, *Ae. albopictus* formed a clade with populations from Thailand, the USA, and Cameroon with 100% bootstrap value, while *Ae. albopictus* populations from China, Brazil, Albania, Italy, France, Taiwan, Mexico, and Japan formed separate clusters. Interestingly, the Philippine *Ae. albopictus* isolate diverged from other populations, suggesting genetic heterogeneity that warrants further investigation. Additionally, our phylogenetic tree identified some additional findings. *Ochlerotatus vigilax* from Brazil and Australia formed a separate sister clade (100%) due to their mitogenome sequence peculiarities, while *Ae. bucki* and *Ae. notoscriptus* from Saba and Australia were their close relatives. *Ae. japonicus* from the USA and Japan were also close related as they formed a separate lineage with strong support (100%). The *Ae. vexans* populations from China and the USA clustered together with a bootstrap value of 100%, indicating a close evolutionary relationship. The outgroup species *C. quinquefasciatus* formed a distinct clade, reinforcing its distant evolutionary relationship within the Culicidae family.

## Discussion

The findings of this study provide insights into the mitogenomic characteristics of *Ae. aegypti* and *Ae. albopictus* from Bangladesh, reinforcing their genomic similarities and differences. By sequencing and characterizing these mitogenomes, we have significantly expanded the genomic resources available for these major dengue vectors in this region, especially Southeast Asia. Our findings reveal distinct genetic features and variations in the mitochondrial DNA of both species, providing valuable insights into their evolutionary trajectories and population genetics. This study represents the first comprehensive analysis of the complete mitochondrial genomes of *Aedes aegypti* and *Aedes albopictus* collected from Bangladesh, revealing a high degree of similarity with previously studied *Aedes* species. The high sequence similarity (>99%) between the newly sequenced mitogenomes and reference sequences confirms the consistency of mitochondrial genome structures across different populations.

The genomes of both species contain all 37 genes, including 13 protein-coding genes (PCGs), and exhibit AT biasness. The observed AT-rich composition aligns with known insect mitogenomic trends, supporting the influence of replication-associated biases. The bias resembles the presumed distinct features of previously recorded mosquito mitochondrial genomes, such as *Ae. aegypti* (AT: 78.2%), *Ae. vexans* (AT: 78.54%), and *Ae. fluviatilis* (AT: 77.5%) [36–38]. Insect mitogenomes display a unique strand-specific nucleotide composition bias, resulting from mutations associated with replication or transcription [39]. A high proportion of AT pairs suggests that most of the mitogenome backbone is composed of adenine and thymine, compared to the proportions of guanine and cytosine. Our findings output similar results of the other insect family Culicidae where the overall AT skew was 77.08% [40].

Our in-depth analysis of mitogenomes showed some unique features between two Aedes species. Among the 38 genes (13 PCG, 2rRNA, 22 tRNA and 1 CR) of both species, the strand-ness and sizes are mostly similar, except strandness of *trnS1* and *trnL1* and minor base pair differences of 18 genes (*trnI, trnQ, trnM, trnY, trnL2, trnK, nad3, trnR, trnA, trnN, trnE, trnT, nad6, cytb, nad1, 12S, 16S* and *CR*). As we used identical processing, assembly and annotation steps, such discrepancy might be attributed to the species-specific changes, which is evident in similar studies [26,41]. In congruent with other mitogenomes from Culicidae, *CR* of both species is located between the *12S* and tRNA-Ile genes which have varying sizes, with a length of 1,721 bp and 1,694 bp, respectively. The CR of the current two species were distinct from others such as *Ae. vexans* (948 bp), *Ae. alboannulatus* (1,072 bp), and *Ae. notoscriptus* (939 bp) [36]. The AT-rich control region typically contains numerous homopolymer sequences and exhibits high variability in both sequence length and mutation rates, which pose challenges for next-generation sequencing technologies [42,43]. It is often difficult to assemble, due to their highly repetitive homology, and often long read sequencing is recommended to resolve the ambiguities. Overall, the control regions exhibit unique sequence and structural features that may be Genus-specific, making them valuable genetic markers for evolutionary and population genetic studies of mosquito species [44]. In the mitochondrial genomes of invertebrates, the most frequent standard initiation codons are found to be ATA, ATG, ATT, and GTG, and the majority of mosquito protein-coding genes use ATG. However, across some species, GTG was observed as the start codon for nad5 and is likely to be a standard feature [45]. In contrast, both *cox1* genes utilize a TCG codon, an unconventional start codon for mitochondrial genes. Comparative analyses with other mosquito mitogenomes indicate that this atypical start codon is also present in beetle (*Thalassa montezumae*) and *Anopheles* (*A. gambiae, A. funestus*) species [45–47], suggesting that it is evolutionarily conserved rather than species-specific. The preference for ATG start codons and incomplete stop codons (T or TA) further reflects the adaptation of mitochondrial translation mechanisms.

A comparative analysis between RSCU values of two species indicates identical features. Codon usage bias highlights evolutionary pressures shaping mitochondrial genomes. Serine and Leucine are the highest used codons in both species, notably the TTA codon of Leucine – which achieved an RSCU value greater than 4. One study [48] showed different arthropods differentially bias their usage of Serine, Arginine and Leucine codons and among which Serine bias is correlated with nucleotide topologies. Overall, similar codon usage and RSCU values are observed in both *Aedes* species, which is identical not only across *Aedes* genera, but also across diverse arthropod taxa [49]. Some minor species specific RSCU values and codon utilization are also observed, highlighting minor evolutionary divergences between the two species, aligning with previous studies on relative synonymous codon usage (RSCU) in *Aedes* species [36,37].

Our comparative assessment of 15 mitochondrial genes, based on K2P and base pair per SNP, showed higher genetic distance, but low SNPs per base pair in *Ae. aegypti* than *Ae. albopictus*. This pattern can be explained by the broader geographic spread and greater genomic diversity among the *Ae. aegypti* specimens analyzed (Australia, Trinidad-Tobago, the United Kingdom, Brazil, and Bangladesh). The higher genetic distance reflects accumulated divergences across these geographically distant populations, while the lower SNP density per base pair may point to past demographic events such as selective sweeps or historical bottlenecks that reduced standing variation. In contrast, *Ae. albopictus* exhibited higher SNP density but comparatively lower genetic distance, consistent with a rapid and more recent global expansion, where high levels of polymorphism are maintained within relatively less divergent lineages. A *coxI-based* study also showed similar trends of higher haplotype diversities of *Ae. aegypti* and better capability to adapt to rapidly changing environmental variabilities [50]. A whole genome wide study among 27 populations of *Ae. aegypti* and *Ae. albopictus* found high genetic distance in *Ae. aegypti* populations, especially in Pacific populations [51]. While we were limited by the accessions available at NCBI, we still found some indication of higher genetic distance of Oceania population (Australia) with that of the specimen from other countries (United Kingdom, Brazil, and Bangladesh). Interestingly, even mitogenomes from twelve different countries were considered for *Ae. albopictus* analysis, a lesser genetic distance was observed with uniform mutational diversities across the genes. This confers to recent population expansion of *Ae. albopictus* in various parts of the world [52]. Together, these findings highlight distinct evolutionary trajectories between *Ae. aegypti* and *Ae. albopictus*, suggesting that demographic history and dispersal patterns have shaped their mitochondrial diversity differently.

The phylogenetic analysis confirmed that *Ae. aegypti* and *Ae. albopictus* form distinct clades, reflecting their evolutionary divergence. Bangladeshi *Ae. aegypti* clustered with populations from Australia and Trinidad-Tobago (bootstrap 96–100%), suggesting either human-mediated dispersal facilitated by global trade and travel or retention of ancestral lineages, while other populations, such as those from the UK and Brazil, formed separate clades. *Ae. albopictus* from Bangladesh grouped with Thailand, USA, and Cameroon populations (bootstrap 100%), whereas isolates from China, Brazil, Europe, Taiwan, Mexico, and Japan formed distinct clusters. The divergence of the Philippine isolate (bootstrap 81–95%) highlights potential local adaptation or unique demographic history. Notably, we could not find mitogenomes of *Ae. albopictus* from Australia (which invaded in 2004) and Trinidad-Tobago (first reported in 2002). Investigating these populations further may provide insights into genetic variations and incursions from neighboring countries. Combined with the published mitogenome sequence of Culicidae, the phylogenetic tree confirmed the monophyly and position of Culicidae with high support value. Our phylogeny placed members of *Aedini* tribe together, which consists of four closely related taxa, namely *Aedes*, *Ochlerotatus*, *Haemagogus*, and *Psorophora* genera. The topology placed *Aedes aegypti* closer to *Ae. vexans,* which is well supported by previous studies [36]. Members of *Ae. albopictus* appeared separately as a cluster and closely appeared with its sister species, *Ae. flavopictus*. *Ae. flavopictus* is often a rare species, which often co-occurs with *Ae. albopictus* [53]*.* The members of the Culicini tribe (*Culex* spp.) are used as an outgroup and well separated from the *Aedini* tribe*.* A molecular study showed these tribes diversified approx. 130 million years ago [38,54].

Overall, these findings contribute valuable genetic data for mosquito evolutionary studies and provide a foundation for understanding mitochondrial genome variability within *Aedes* species. Although current study has got some limitations, e.g., single individual sequencing, lack of long read-based sequencing data, these short-read based mitogenome assemblies still play valuable role to trace invasion routes of these notorious vectors and also can be instrumental to track presence of insecticide resistance. The data may also facilitate vector control strategies by elucidating genetic markers for population differentiation and transmission dynamics. The detailed mitogenomic sequences obtained offer a robust framework for further studies on the genetic diversity and adaptation of these mosquitoes. The identification of specific mitochondrial gene variants and potential markers of interest enhances our understanding of the molecular basis underlying vector biology and disease transmission dynamics in Bangladesh. This baseline genomic data marks a significant advancement in our ability to combat several human arboviral diseases especially Dengue, Chikungunya, Yellow fever, Zika through genomics-informed vector management approaches.

## Supporting information

S1 Fig AT/GC contents and AT/GC-skews of the investigated mitogenomes. (TIF)

S2 Fig Identity similarity matrix of mitogenomes of different mosquito species. Sample information and accessions can be found in supplementary S1 Table.
(TIF)

S3 Fig Bayesian inference of phylogenetic tree based on concatenated PCGs of different mosquito species. Bayesian posterior probability values are shown in nodes.
(TIF)

S1 Table A table showing list of mosquito sequence accessions utilized for comparative study. (PDF)
