# Supplementary figures and images for: Complete mitogenomes of two major dengue vectors Aedes aegypti and Aedes albopictus from Bangladesh: Insights from comparative genomics with global mitogenome diversity and phylogenetics
Source: PLoS One. 2025 Sep 30;20(9):e0333693. doi: 10.1371/journal.pone.0333693 (PMC12483262; doi:10.1371/journal.pone.0333693)

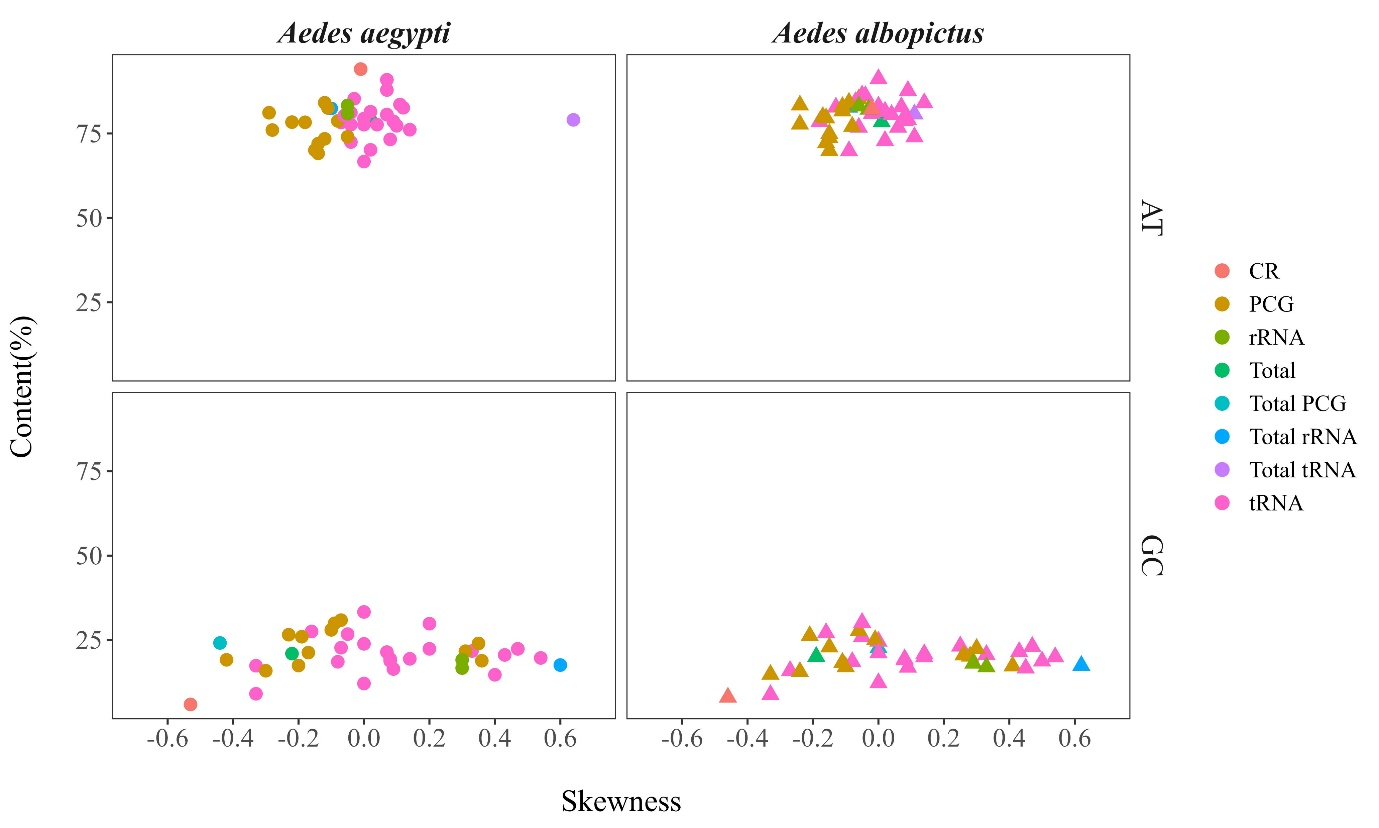

Supplement: S1 Fig — (TIF) [file pone.0333693.s001.tif]

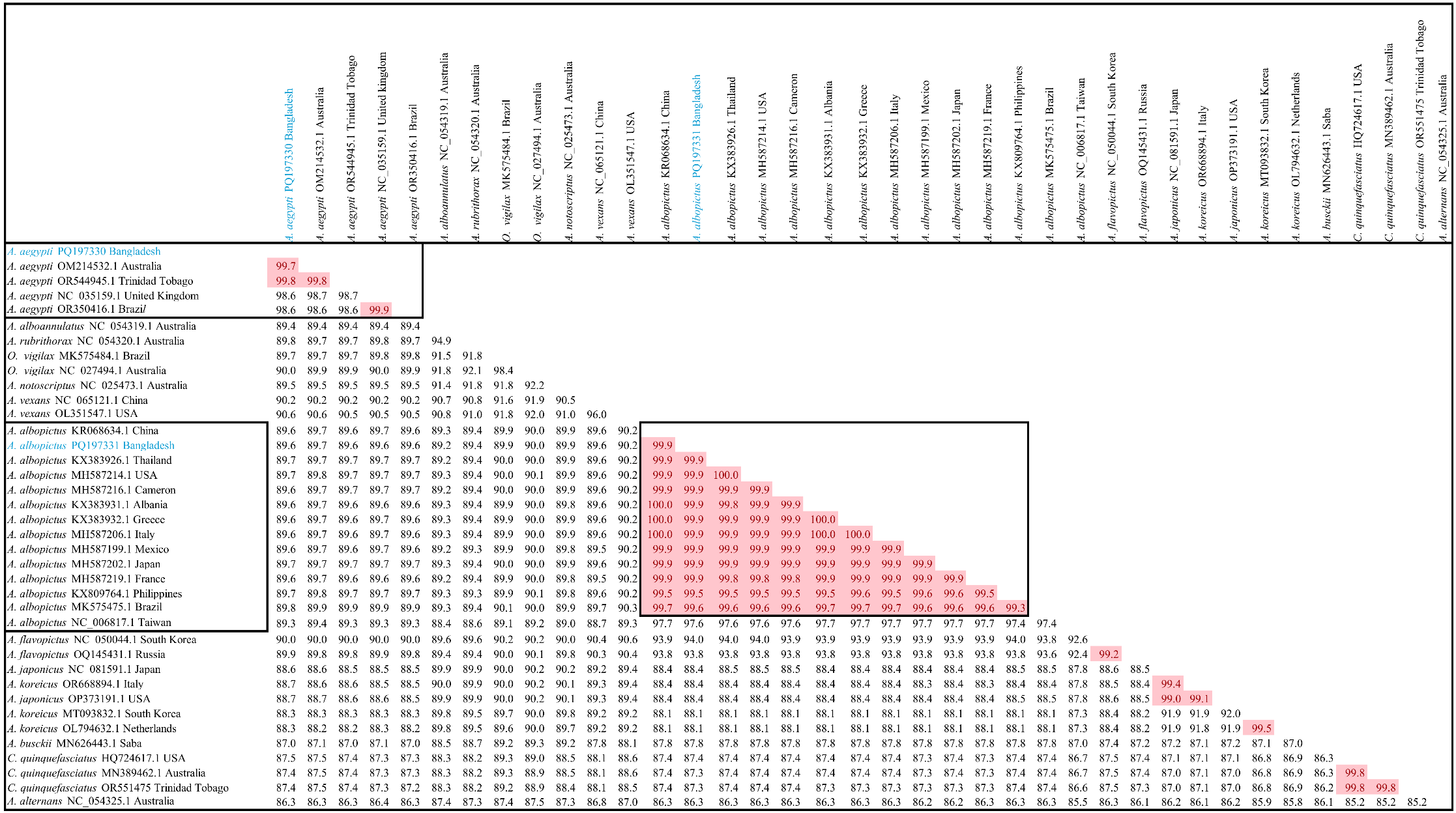

Supplement: S2 Fig — Sample information and accessions can be found in supplementary S1 Table. (TIF) [file pone.0333693.s002.tif]

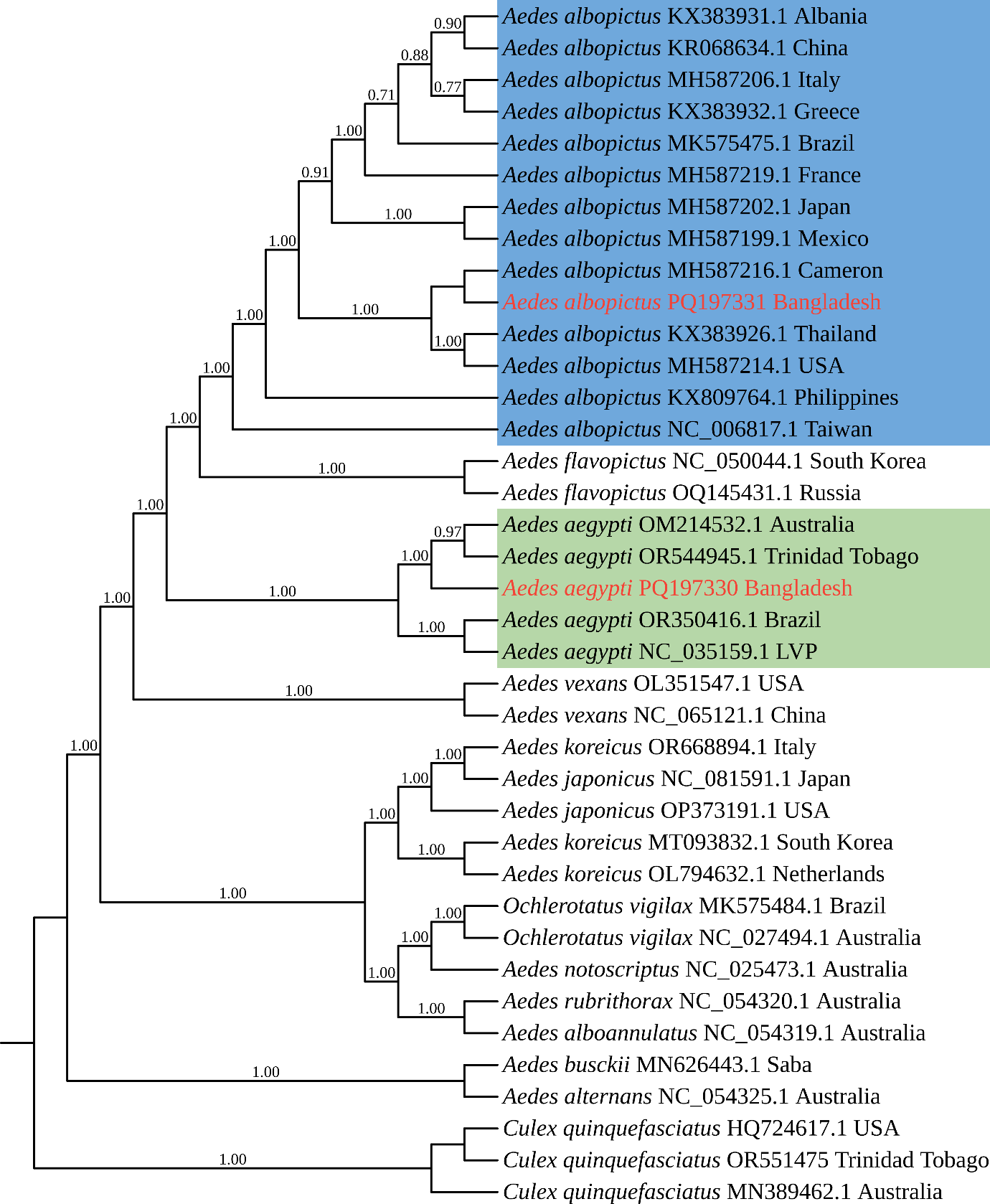

Supplement: S3 Fig — Bayesian posterior probability values are shown in nodes. (TIF) [file pone.0333693.s003.tif]
