# Supplementary material for: Complete mitogenomes of two major dengue vectors Aedes aegypti and Aedes albopictus from Bangladesh: Insights from comparative genomics with global mitogenome diversity and phylogenetics
Source: PLoS One. 2025 Sep 30;20(9):e0333693. doi: 10.1371/journal.pone.0333693 (PMC12483262; doi:10.1371/journal.pone.0333693)
Supplement: S1 Table — (PDF) [file pone.0333693.s004.pdf]

Table S1. A table showing list of mosquito accessions utilized for comparative study.

Species	Accession Number	Country
Aedes albopictus	KR068634.1	China
Aedes albopictus	MK575475.1	Brazil
Aedes albopictus	KX383931.1	Albania
Aedes albopictus	KX383932.1	Greece
Aedes albopictus	MH587206.1	Italy
Aedes albopictus	MH587219.1	France
Aedes albopictus	NC_006817.1	Taiwan
Aedes albopictus	MH587199.1	Mexico
Aedes albopictus	MH587202.1	Japan
Aedes albopictus	KX383926.1	Thailand
Aedes albopictus	MH587214.1	USA
Aedes albopictus	MH587216.1	Cameroon
Aedes albopictus	PQ197331	Bangladesh
Aedes albopictus	KX809764.1	Philippines
Aedes flavopictus	NC_050044.1	South Korea
Aedes flavopictus	OQ145431.1	Russia
Aedes aegypti	OM214532.1	Australia
Aedes aegypti	OR544945.1	Trinidad Tobago
Aedes aegypti	PQ197330	Bangladesh
Aedes aegypti	NC_035159.1	United Kingdom
Aedes aegypti	OR350416.1	Brazil
Aedes vexans	NC_065121.1	China
Aedes vexans	OL351547.1	USA
Aedes japonicus	OP373191.1	USA
Aedes japonicus	NC_081591.1	Japan
Aedes koreicus	MT093832.1	South Korea
Aedes koreicus	OL794632.1	Netherlands
Aedes alternans	NC_054325.1	Australia
Aedes alboannulatus	NC_054319.1	Australia
Aedes rubrithorax	NC_054320.1	Australia
Aedes notoscriptus	NC_025473.1	Australia
Aedes busckii	MN626443.1	Saba
Ochlerotatus vigilax	MK575484.1	Brazil
Ochlerotatus vigilax	NC_027494.1	Australia
Culex quinquefasciatus	OR551475	Trinidad Tobago
Culex quinquefasciatus	MN389462.1	Australia
Culex quinquefasciatus	HQ724617.1	USA
